# Behavioral Effects of Low Concentrations of Boric Acid and Thiamethoxam Baits on the Laboratory Colonies of the Argentine Ant

**DOI:** 10.3390/insects17080785

**Published:** 2026-07-29

**Authors:** DongChan Im, Daniel Dar, Vincent Le, Wenbo Li, Dong-Hwan Choe

**Affiliations:** 1 Department of Biology, University of California, Riverside, CA 92521, USA; chan.im@email.ucr.edu (D.C.I.); vincent.le1@email.ucr.edu (V.L.); 2 Department of Microbiology, University of California, Riverside, CA 92521, USA; daniel.dar@email.ucr.edu; 3 Department of Entomology, University of California, Riverside, CA 92521, USA; wli295@ucr.edu

**Keywords:** activity, clumping, neonicotinoid, boric acid, sublethal effect

## Abstract

The Argentine ant, *Linepithema humile* (Mayr), is an important invasive pest in natural, agricultural, and urban ecosystems worldwide. Boric acid and thiamethoxam have been used as bait toxicants for Argentine ant management. Most of the toxicological investigations on these toxicants have focused on the mortality of the ants. However, their behavioral effects on target ants remain poorly understood. By focusing on the in-nest aggregation and out-nest activity level, the current study investigated the behavioral effects of low concentrations of boric acid and thiamethoxam on Argentine ants. Boric acid increased in-nest aggregation and reduced activity in the foraging area. In contrast, thiamethoxam treatment caused the ants to leave the nest and continue roaming in the foraging area. Individual behavior assay showed that overall mobility of ants was not impacted by any of the treatments, while thiamethoxam treatment caused ants to spend more time in the lighted area. Distinct behavioral impacts of these toxicants might be due to differences in their unique modes of actions. Understanding these behavioral effects of bait toxicants would help to optimize management protocols for the Argentine ant.

## Simple Summary

The Argentine ant is an invasive pest commonly found in urban, agricultural, and natural environments. Toxic baits are widely used to manage this species, but little is known about behavioral impacts of bait toxicants on the ants. To simulate the situations where bait toxicants get diluted via trophallaxis (mutual food sharing), laboratory colonies of Argentine ants were treated with sucrose water baits containing small amounts of boric acid or thiamethoxam. Density of ants within the nest and colony’s activity level in the foraging area were recorded over time. Boric acid treatment resulted in increased aggregation within the nest and suppressed out-nest activity. In contrast, thiamethoxam treatment caused the ants to leave the nest and continue roaming outside of the nest, significantly increasing their activity level in the foraging area. Even though overall mobility of ants was not impacted by any of the treatments, thiamethoxam treatment affected the ants’ response to light. The findings demonstrate these bait toxicants’ unique behavioral impacts on Argentine ants.

## 1. Introduction

The Argentine ant, *Linepithema humile* (Mayr) (Hymenopteran: Formicidae), was introduced to the US from South America in the late 19th century and has since become a common pest ant species in some southern US states, California, Nevada, Arizona, and Texas [1]. It is also found in the Mediterranean, Southern Africa, Japan, and Oceania [1]. Their adaptability to human-built environments, combined with their competitive behavior and ability to form supercolonies, contributes to their pest status in urban settings [2]. Argentine ants are also considered a significant pest in agricultural and natural settings due to their symbiotic relationship with honeydew-producing plant pests [3]. Additionally, their high foraging efficiency and aggressive nature leads to competitive displacement of some native ants and other arthropods [4]. This can lead to declines in native biodiversity and disruption of natural ecosystem function in the invaded habitats.

Various insecticidal baits have been tested to manage Argentine ant populations. Since foraging Argentine ants readily accept aqueous bait solutions containing sucrose, liquid baiting is an effective method for Argentine ant control [5]. For liquid baiting, the insecticidal compound (i.e., active ingredient) should be soluble in water [6]. Delayed action (i.e., slow kill over 3–4 days) and absence of feeding deterrence are also among the important requirements for liquid baiting [6]. Furthermore, an effective bait toxicant should remain effective even after the liquid bait becomes diluted through trophallaxis (i.e., food sharing) among different members of the colony [7].

Two active ingredients, boric acid (and borate salts) and thiamethoxam, have been widely used for Argentine ant baiting. At the concentrations typically used in bait products, these compounds meet the aforementioned requirements for an effective bait toxicant [8]. Boric acid is an inorganic compound that has been used to control insects such as cockroaches and ants [9]. Boric acid and related borate salts primarily work as stomach toxins [10]. Klotz et al. reported that a 25% sucrose solution with a range from 0.5 to 1% (wt/vol) boric acid was most effective against Argentine ants, providing an optimal palatability and mortality outcome [11]. Recently, boric acid has also been used as a bait toxicant in a hydrogel bait (at 1%) to control Argentine ants [12,13]. Thiamethoxam is a neonicotinoid insecticide that works as an agonist on nicotinic acetylcholine receptors (nAChRs) in insect nervous systems [14]. Thiamethoxam is an effective bait toxicant for Argentine ant control at concentrations of 0.01–0.0001% in aqueous sugar baits [15,16]. When used in the form of hydrogel baits, 0.007–0.0006% thiamethoxam was effective at suppressing Argentine ant populations in the field [8,17,18].

Most bait studies assess two parameters to determine the efficacy of baiting: mortality and foraging activity. Number of dead ants or percentage mortality is often used to measure bait efficacy in laboratory experiments [6]. For field experiments, foraging activity was used to determine the efficacy of baiting [6,19]. The foraging activity of ants in the field can be estimated based on the number of ants or their visits at monitoring sites or stations [13,15,20]. Often, the reduction in foraging activity is considered an indication of positive bait efficacy due to mortality caused by baiting.

In contrast, behavioral impacts of boric acid and thiamethoxam on Argentine ants (as bait toxicants) are not well understood. Considering their slow action and further dilution through trophallaxis, it is reasonable to assume that large portions of the baited Argentine ant population would be exposed to these insecticides at sublethal doses [6]. Are the sublethal behavioral effects, at least in part, responsible for the reduction in foraging activity after baiting? Do the behavioral impacts of these insecticides have any unintended consequences in pest infestation and baiting outcomes? Knowledge on behavioral impacts of these insecticides at low concentrations would be useful to answer these questions.

Behavioral effects of various neonicotinoids on ants have been previously documented [21]. In Argentine ants, sublethal exposure to a neonicotinoid, imidacloprid, in bait did not affect their foraging behavior (e.g., food discovery probability), but it increased the level of their aggressiveness in interspecific confrontations [22]. Our preliminary observation with laboratory colonies of Argentine ants indicates that treatment with thiamethoxam bait made the ants leave the nest and constantly wander in the foraging areas before dying [23]. On the other hand, the behavioral effects of boric acid ingestion have not been previously explored. Ingested boric acid causes irreversible damage to the epithelial cells of the Malpighian tubules and midgut in various insects, including ants [24,25,26]; this cellular damage can impair water balance and detoxification processes in some social hymenopterans [25]. In Florida carpenter ant, *Camponotus floridanus* (Buckley), boric acid baiting caused the ants to remain on the cotton plugs of the bait vials for longer periods of time, possibly due to a disruption of water regulation [27]. Our preliminary observation showed that laboratory colonies of Argentine ants treated with boric acid bait tend to aggregate within their nesting sites, closer to a water source [23,28].

The current study aims to characterize behavioral effects of boric acid and thiamethoxam on Argentine ants when used as a bait toxicant. To simulate the dilution of toxicants via trophallaxis, the toxicants were incorporated in the bait at sublethal concentrations (i.e., most of the treated ants remained alive during the entire experimental period—4 days). The experimental arena was designed to allow for the observation of Argentine ant colonies inside of the nest as well as in the foraging area. For the in-nest behavior, the clumping (aggregation) behavior was quantified based on the volumetric density of ants within the nest. For the out-nest activity, the number of ants in the foraging area were recorded. Lastly, behavioral tracking assays were conducted to investigate whether the treatment had any effect on ant mobility in a dark/light environment. By investigating these in-nest aggregations and out-nest activities, the current study sought to elucidate the broader impact of boric acid and thiamethoxam baiting on the Argentine ant colony, beyond just mortality. Alterations in these behaviors may indicate disruptions in physiological or neurological functions by the insecticides. The findings may provide important information regarding changes in ant activity in the field following the use of the baits containing these active ingredients.

## 2. Materials and Methods

**Ants.** Argentine ants were collected by digging up ant nests in citrus groves located on the University of California, Riverside, campus and placing them into 19 L buckets. The ants were separated from soil by methods described by Hooper-Bui and Rust [29], and placed in a larger colony box (31 by 77.5 cm) with plastic Petri dishes filled with plaster (DAP Plaster of Paris Dry Mix; DAP Inc., Baltimore, MD, USA), which served as nests. The sides of the colony box were coated with fluoropolymer resin (Insect-A-Slip—Fluon^®^; BioQuip Products, Inc., Rancho Dominguez, CA, USA) to prevent the ants from escaping. Wrinkled paper towels were also provided as additional living space for ants. Water, pieces of canned chicken breast (Swanson Premium Chunk Chicken Breast, Campbell Soup Co., Camden, NJ, USA), and 25% (wt/vol) sucrose water were provided ad libitum. The stock colonies were held at 26–27 °C and 30% relative humidity (RH).

**Figure 1:**
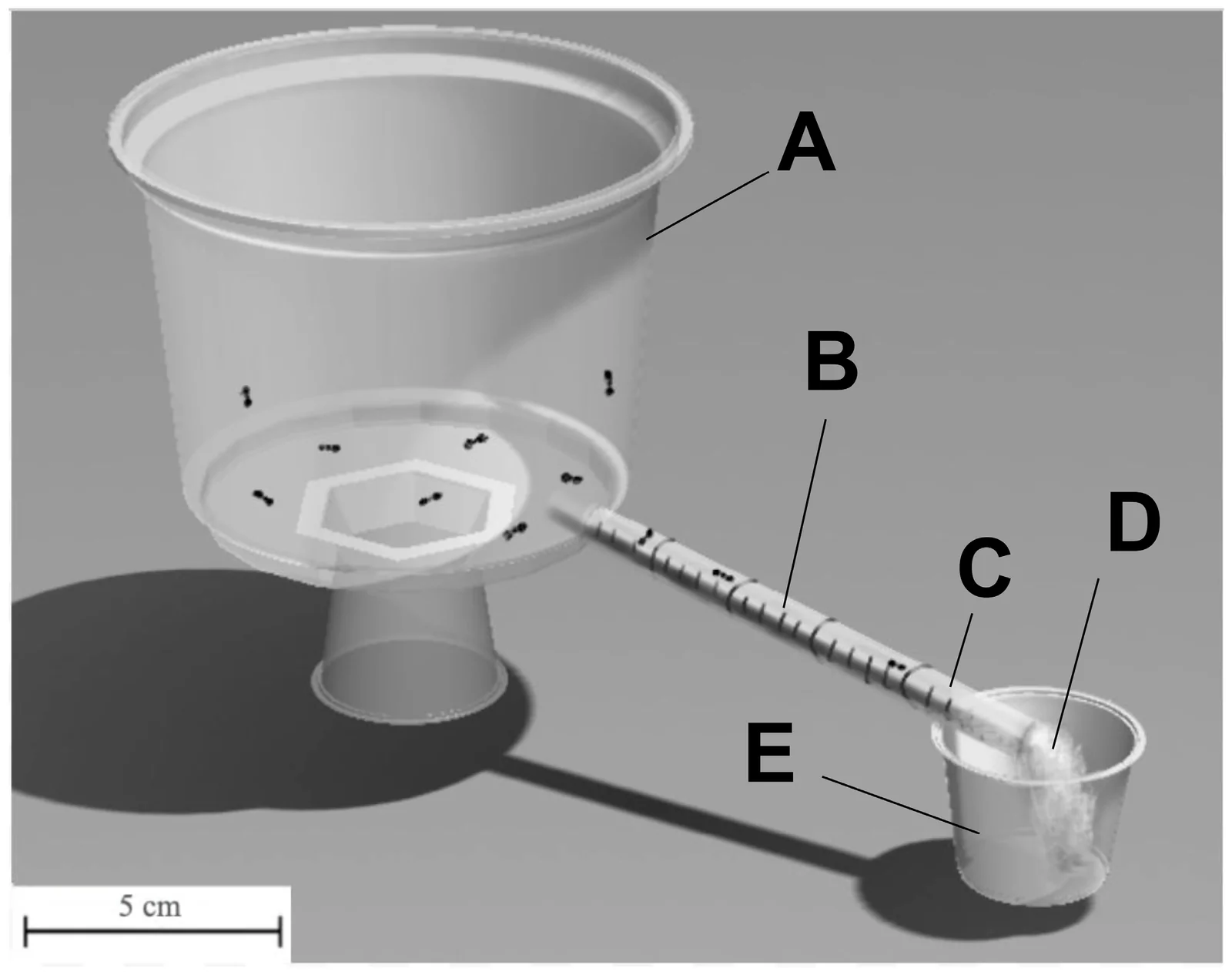
A digital rendering of the experimental arena. The main foraging area (out-nest) (**A**) was connected with the nesting tube (in-nest) (**B**), where moisture was provided from the plaster plug (**C**). A piece of cotton wick (**D**) drew water from a water reservoir (**E**) to the plaster plug by capillary action.

**Experimental arena.** An experimental arena was designed to allow the observation and quantification of the behaviors of Argentine ants in their nest (in-nest) as well as foraging area (out-nest). The arena had three main components—a nesting tube, a moisture source for the nesting tube, and a foraging area (Figure 1). A 4.5 mL section (1 cm in diameter) of a 10 mL polystyrene pipet (Fisherbrand™ Sterile Polystyrene Disposable Serological Pipets with Magnifier Stripe, Fisher Scientific, Waltham, MA, USA) served as the nesting tube (B in Figure 1). A short cylindrical plug (about 0.5 mL in volume) of plaster was formed inside of one end of the nesting tube (with 0.5 mL room at the end for the cotton wick, see below), providing a physical barrier which limited the movement of ants while allowing water to permeate from outside (C in Figure 1). A 7 cm section of cotton wick (Braided Cotton Roll, Medium, ⅜” × 6”; Richmond Dental. Charlotte, NC, USA) was inserted into the nesting tube, contacting the plaster plug (D in Figure 1). A 30 mL plastic cup was attached to one end of the nesting tube with hot glue (Crystal Clear ™ Premium Bond Glue; Adhesive Technologies, Inc., Hampton, NJ, USA) and filled with water, serving as a water reservoir (E in Figure 1). The end of the cotton wick was placed in the plastic cup, drawing water to the plaster plug inside the nesting tube. This design allowed a continuous and controlled supply of small amounts of moisture into the nesting tube. In addition, since the ants were unable to chew or dig through the plaster plug, this experimental nest design provided a fixed space, 3.5 mL in volume, as a nesting site for the experimental colony.

A 480 mL plastic cup (First Street Deli Containers, Clear Plastic, 16 Ounce; Smart & Final, Los Angeles, CA, USA) served as the foraging area (A in Figure 1). The inner wall of the cup was coated with fluoropolymer resin (Insect-A-Slip—Fluon^®^; Bioquip Products, Inc., Rancho Dominguez, CA, USA) to prevent the ants from escaping. A hole (1 cm in diameter) was made on the wall near the bottom of the foraging area with a soldering iron, and the nesting tube was inserted until 0.5 mL of the total 4.5 mL volume was within the foraging area. The nesting tube was then secured in position with hot glue. The position of the foraging area was slightly elevated above the water reservoir (by 1.0 cm), creating an incline to prevent flooding inside the nesting tube. A cap from a microcentrifuge tube (Fisherbrand™ Microcentrifuge Tubes with Locking Snap Cap; Thermo Fisher Scientific, Waltham, MA, USA), placed upside down in the bottom of the foraging area, served as a feeding dish.

Four days before bait application, the following method was used to set up the experimental colonies. Ants from the stock colony were transferred into a smaller, empty plastic bin (34 × 20 × 6 cm). Several 2 cm sections of clear plastic tubing (Tygon^®^ S3™ E-3603 Laboratory Tubing; Saint-Gobain Performance Plastics Corp., Akron, OH, USA) were then placed in the plastic bin. Once the ants (workers and queens) moved into these tubing sections, each of them was gently picked up and the ants were tapped into the foraging area of the arena. This process allowed us to prepare multiple experimental colonies of comparable size and composition. In the current experiment, the average number of ants per experimental colony, excluding queens, was 294.2 ± 12.3 (mean ± SEM, *n* = 30). All colonies had similar amounts of brood and eggs, and most of the colonies had between 1 and 3 queens.

**Bait.** For boric acid (BA), 0.2% (wt/vol) bait was prepared by dissolving boric acid [ReagentPlus^®^ [9]; Sigma-Aldrich, St. Louis, MO, USA] in 25% (wt/vol) sucrose water. For thiamethoxam (THX), 0.00005% (wt/vol) bait was prepared by dissolving thiamethoxam [PESTANAL^®^ (99.7%), analytical standard; Sigma-Aldrich, St. Louis, MO, USA] in 25% sucrose water. These concentrations were chosen based on preliminary trials where clear behavioral effects in Argentine ant colonies were observed without substantial mortality up to day 3 (e.g., <10 dead ants) [23]. Klotz et al. reported that 0.2% boric acid in sucrose baits provided delayed toxicity with lethal times exceeding 5 d [30]. Tay et al. showed that 0.00001–0.00004% thiamethoxam bait delivered (in calcium alginate hydrogels) to the laboratory colonies of Argentine ants provided day 3 mortality which was not significantly different from that of untreated control [18]. A 25% sucrose water solution served as a control (CTRL).

**Bait application and data collection.** All colonies in experimental arenas were allowed to acclimate for a day before being fed with 10 μL of sucrose water (25% wt/vol) provided in the feeding dish. Bait solutions (10 μL) were administered 3 d after the initial feeding of the sucrose water, and this date was referred to as day 0. The bait solutions were provided only once (on day 0) in a no-choice setting (i.e., no other food sources were available). Using an iPhone 13 (Apple Inc., Cupertino, CA, USA; 1080p resolution), the nesting tube (side view) and the entire foraging area (top view) were photographed immediately before the bait application (day 0), and after the bait application, every 24 h for 3 d (day 1–3). These images were used to quantify the in-nest aggregation and out-nest activity (see below). Subsequently, all experimental colonies were euthanized by first CO_2_ anesthetization and freezing in −18 °C for 24 h. Following euthanization, the ants were thawed, spread out on individual sheets of paper, and manually counted to determine the total number of ants in each colony. Eggs and brood were not considered in this study. The control and treatments (boric acid and thiamethoxam) were replicated 10 times (total 30 colonies).

**Figure 2:**
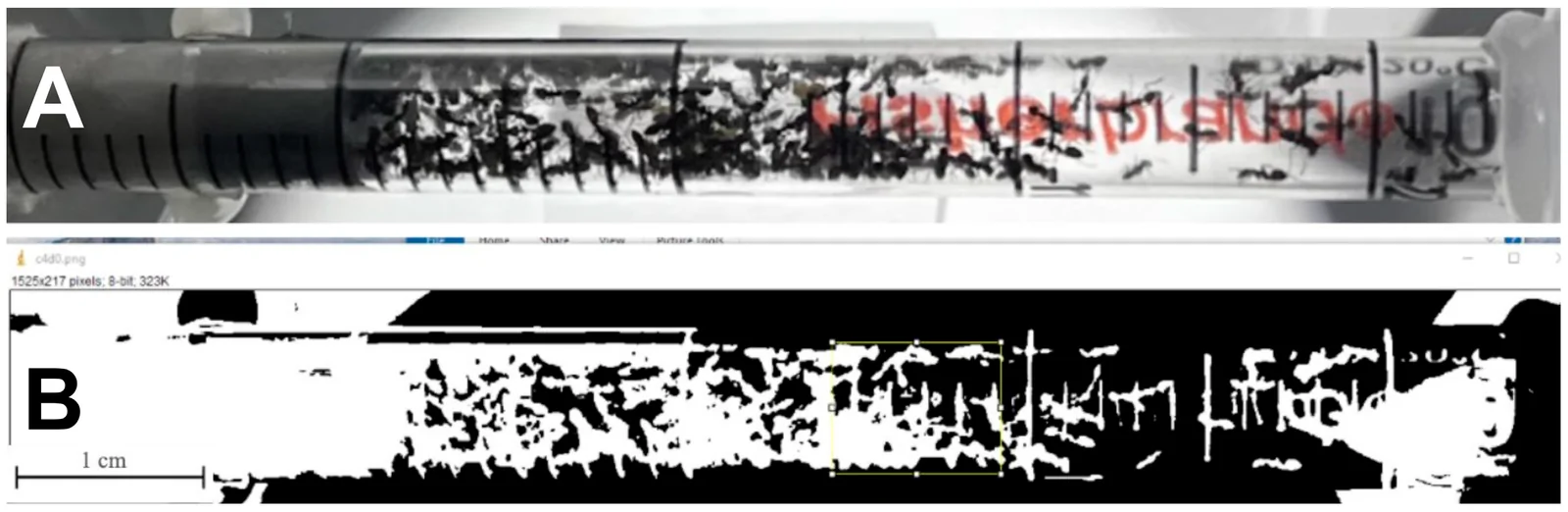
Discriminating ants from their backdrop through ImageJ. Original photo shown above (**A**); processed image shown below (**B**).

**In-nest aggregation and out-nest activity.** For the in-nest aggregation, density of ants inside of the nesting tube was measured. To quantify the density of ants in the nesting tube, the side view images of the nesting tube were analyzed using the Java-based processing software ImageJ (ver. 1.53) [31] (Figure 2). The images were converted to 8-bit grayscale and thresholded to distinguish ant silhouettes from the background (Figure 2B). The area fraction tool was used to determine the point at which ants occupied less than 50% of the visible area in 0.5 mL intervals. This was done by creating a rectangle scanning tool corresponding to 0.5 mL of length in the image and sliding it from the innermost area of the nesting tube towards the nest entrance. Once the value of 50% was digitally observed, the outermost side of the rectangle was used to define a limit line, marking the margin of aggregation within the nesting tube. The 50% threshold was chosen based on preliminary observations of ant clumping behavior and it was consistent with visual limits across images. Because the ants tended to cluster near the water source (innermost area of the nesting tube), volume measurements were taken starting from the plaster plug and extending to the limit line.

The number of ants within this aggregation was calculated by subtracting the number of ants found outside the limit (both in the remaining part of the nesting tube and the foraging area) from the total ant count. Finally, density was calculated using the following formula:

$$density\;in\;aggregation\;(\mathrm{ants}/mL)=\frac{total\;number\;of\;ants\;-\;number\;of\;ants\;outside\;aggregation}{volume\;of\;aggregation\;(mL)}$$

For the out-nest activity, the total number of ants in the foraging area was counted based on the photographs.

**Figure 3:**
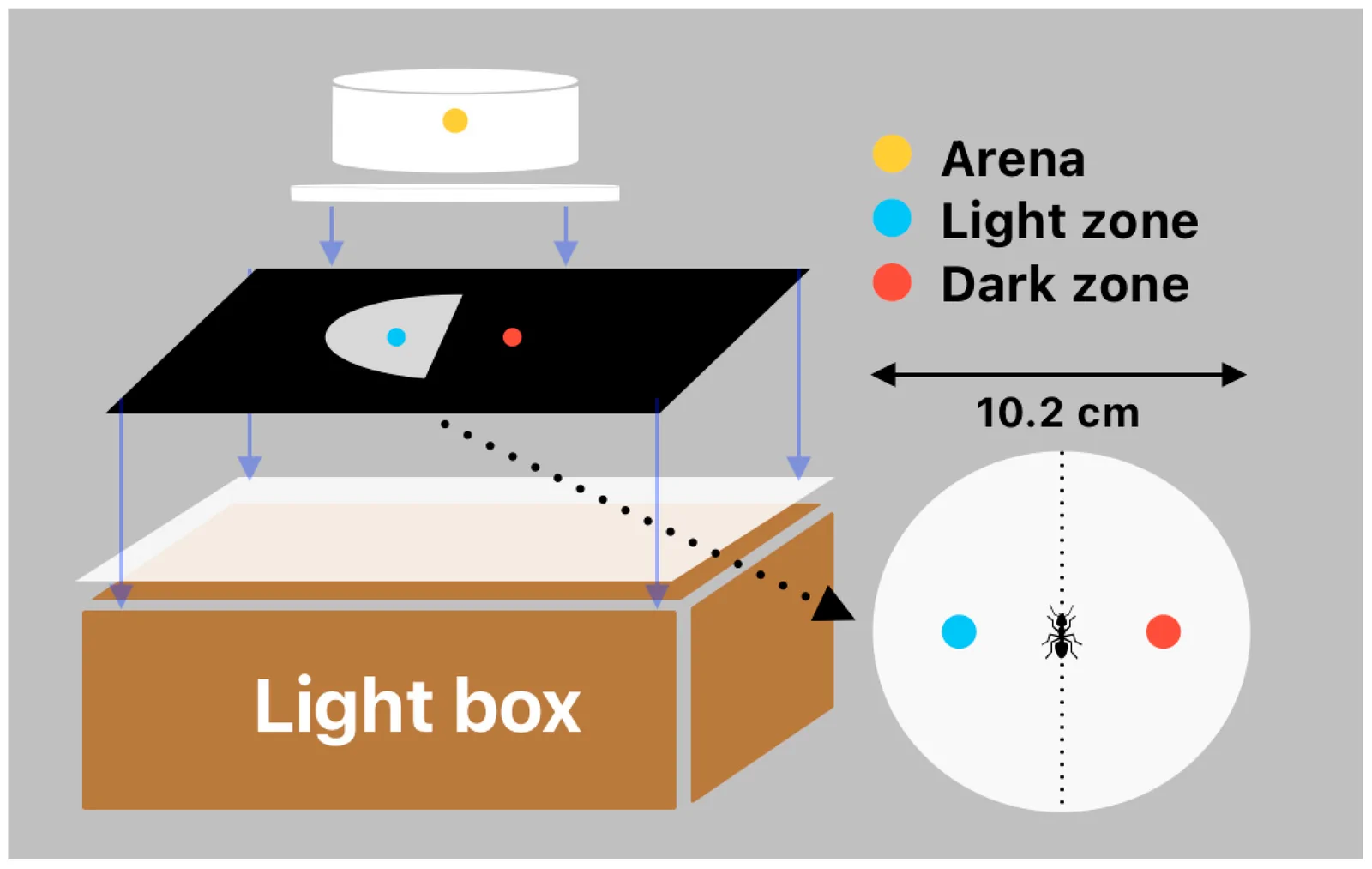
Individual behavior assay arena setup. The circular arena consisted of a glass floor (lined with filter paper) enclosed by a plexiglass ring. A black paper cover with a hemispherical cutout was placed over a light box to create distinct light and dark zones within the circular arena. Individual ants were recorded for 10 min using a near-infrared camera and infrared illumination installed above the arena.

**Individual behavior assay.** Sublethal effects of the bait toxicants on individual ant mobility in the dark/light environment were tested using the Ethovision XT tracking system (Version 11.5, Noldus Information Technology, Wageningen, The Netherlands). The movement of one ant was recorded with a near-infrared camera (Basler, acA1300-60gmNIR, Ahrensburg, Schleswig-Holstein, Germany) in a circular behavior assay arena illuminated with an infrared light source (Axton AT-11 S, 850 nm, 150°, North Salt Lake, UT, USA). The behavior assay arena was prepared by placing a plexiglass ring wall (10.2 cm inner diameter) on a circular glass plate floor (14.2 cm diameter) (Figure 3). The inner surface of the plexiglass ring wall was coated with fluoropolymer resin to prevent ants from escaping. The arena was placed on a UV-free 10,000-lux light box (TRU-VIEW Lightbox, Logan Electric Spec. Mfg. Co., Chicago, IL, USA) covered with a black paper sheet with a hemispherical cutout (10.2 cm diameter). This arena setup created distinct light and dark zones within the circular arena. A 15 cm diameter filter paper disk (Whatman^®^ qualitative filter paper, Grade 1, GE Healthcare UK Ltd., Buckinghamshire, UK) placed between the glass arena floor and plexiglass ring served as the arena floor liner. Experimental colony preparation, bait preparation and treatment followed the same procedure described above. On day 3 after bait treatment, one ant was gently picked up from the experimental colony floor (using a small piece of rolled tissue paper) and introduced into the behavior assay arena. The activity of each ant was recorded for 10 min. The filter paper floor liner was replaced between trials to minimize chemical or pheromone contamination. Each treatment group was replicated 16 times, with a total of 48 trials conducted. Total distance traveled within the arena and total cumulative time spent in the light or dark zone were recorded and compared between the control and the treatments.

**Statistical analyses.** For the density, number of ants within bounds, volume occupied in the nesting tube, and out-nest activity, a Friedman test, a nonparametric alternative to a one-way repeated-measures analysis of variance [32], was used to analyze changes over time (day 0–3) within each treatment group. For the individual behavior assay, data from control and two treatments were compared with a Kruskal–Wallis test. When significant differences were detected, Dunn’s multiple comparisons test (*α* = 0.05) was used to identify which specific groups differed significantly. *p* values were adjusted with Bonferroni correction to control the family-wise error rate. All statistical tests were conducted using Statistix 10.0 [33].

## 3. Results

**Figure 4:**
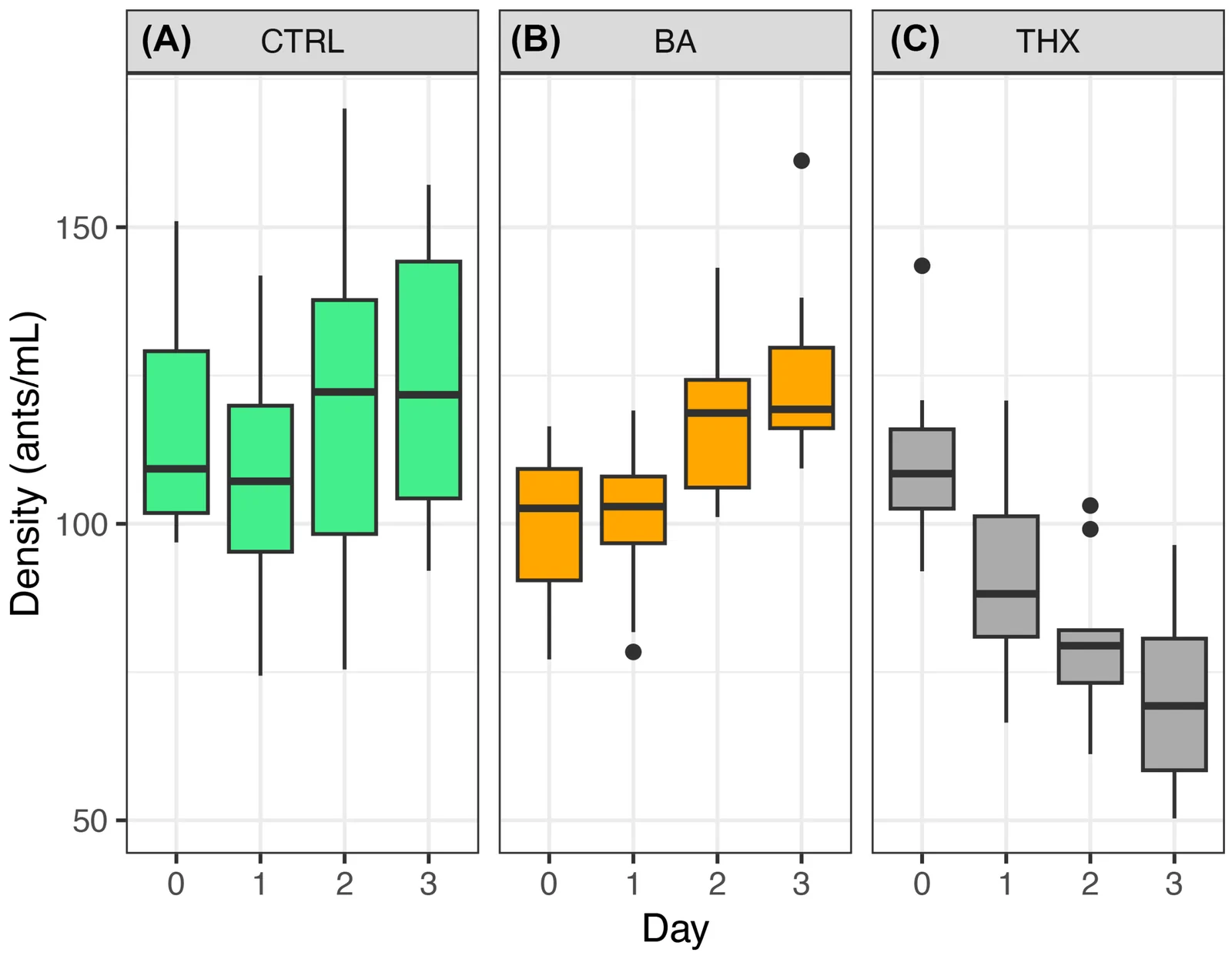
Density of ants (ants/mL; *y*-axis) within nesting tubes measured across the experimental period (day 0, 1, 2, and 3; *x*-axis). Data for day 0 were collected immediately before the bait application. (**A**) Untreated control (CTRL, sucrose water), (**B**) boric acid (BA, 0.2% *w*/*v* in sucrose water), and (**C**) thiamethoxam (THX, 0.00005% *w*/*v* in sucrose water). The center line within each box represents the median; box limits indicate the interquartile range (25th–75th percentiles); whiskers extend to the minimum and maximum values within 1.5× of the interquartile range. Dots represent potential outliers.

**In-nest aggregation behavior.** The CTRL group exhibited no significant change in ant density over time (*χ*^2^ = 6.84, *df* = 3, *p* = 0.077) (Figure 4A). In contrast, BA and THX groups showed significant differences in ant density over time (*χ*^2^ = 19.92, *df* = 3, *p* = 0.0002 for BA; *χ*^2^ = 25.56, *df* = 3, *p* < 0.0001 for THX) (Figure 4B,C).

Based on Dunn’s all-pairwise comparisons test within each treatment, the BA group showed a significant increase in ant density within the nesting tube for day 2 and 3 compared to day 0 and 1, indicating increased aggregation (Figure 4B, Table 1). The THX group showed a significant decrease in density over time, with density on day 3 significantly lower than those for day 0 and 1 (Figure 4C, Table 1).

Analysis of the number of ants within the in-nest aggregation revealed that CTRL colonies remained relatively similar from day 0 to day 3 (*χ*^2^ = 1.87, *df* = 3, *p* = 0.6), while BA colonies showed a significant increase in number of ants within the aggregation (*χ*^2^ = 13.96, *df* = 3, *p* = 0.003) (i.e., day 2 and 3 data were significantly higher than day 0 data). In contrast, the THX group exhibited a significant decline in the number of ants within the in-nest aggregation over time (*χ*^2^ = 28.76, *df* = 3, *p* < 0.0001) (i.e., day 3 data were significantly lower than day 0 and 1 data), indicating a major shift of ant location from the nest to the other areas of the experimental arena (Table 2).

Analysis of the occupied nest volume revealed that the CTRL group remained similar across all four days, showing no significant changes (*χ*^2^ = 4.34, *df* = 3, *p* = 0.227). The BA group displayed only minor fluctuations in volume (*χ*^2^ = 10.19, *df* = 3, *p* = 0.017), without any significant difference between day 0 and day 3. In contrast, the THX group showed a clear decline in occupied nest volume over time (*χ*^2^ = 24.09, *df* = 3, *p* < 0.0001) (i.e., day 3 data were significantly lower than day 0 data), further supporting a major shift of ant location from the nest to the other areas of the experimental arena (Table 3).

**Figure 5:**
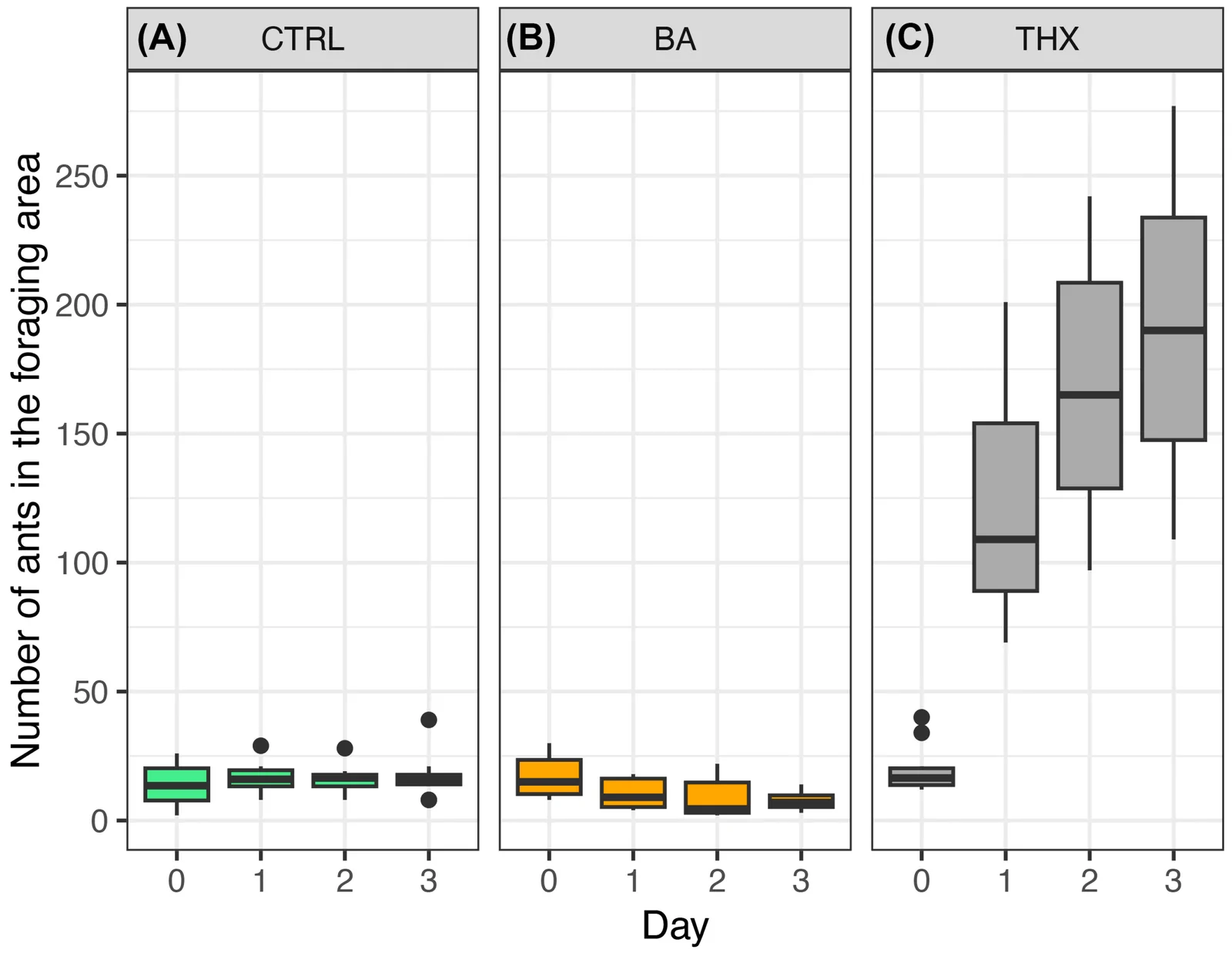
Number of Argentine ants in the foraging area across the experimental period (day 0, 1, 2, and 3; *x*-axis). Data for day 0 were collected immediately before the bait application. (**A**) Untreated control (CTRL, sucrose water), (**B**) boric acid (BA, 0.2% *w*/*v* in sucrose water), and (**C**) thiamethoxam (THX, 0.00005% *w*/*v* in sucrose water). The center line within each box represents the median; box limits indicate the interquartile range (25th–75th percentiles); whiskers extend to the minimum and maximum values within 1.5× of the interquartile range. Dots represent potential outliers.

**Out-nest activity.** The CTRL group exhibited no significant change in ant number in the foraging area over time (*χ*^2^ = 3.58, *df* = 3, *p* = 0.3105) (Figure 5A). In contrast, BA and THX groups showed significant differences in ant number in the foraging area over time (*χ*^2^ = 17.27, *df* = 3, *p* = 0.0006 for BA; *χ*^2^ = 30.00, *df* = 3, *p* < 0.0001 for THX) (Figure 5B,C).

Based on Dunn’s all-pairwise comparisons test within each treatment, the BA group showed a significant decrease in ant number in the foraging area for day 1 through 3 compared to day 0, indicating a reduced out-nest activity (Figure 5B, Table 4). The THX group showed a significant increase in ant number in the foraging area over time, with the data from day 2 and 3 significantly greater than those from day 0 (Figure 5C, Table 4).

**Figure 6:**
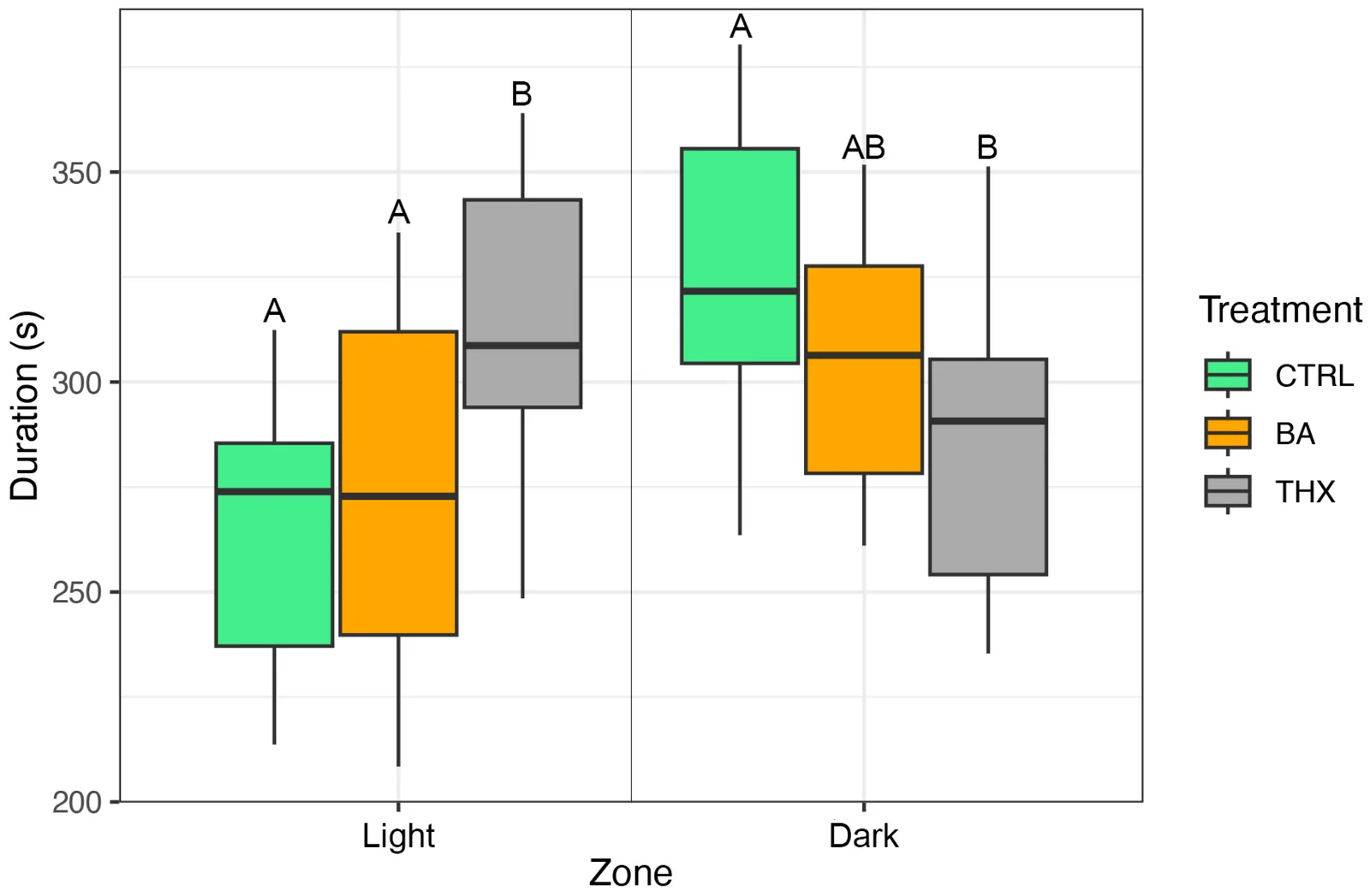
Total time duration (s) spent by an Argentine ant in the light or dark zone (day 3 after treatment) during a 10 min behavioral assay. The center line within each box represents the median; box limits indicate the interquartile range (25th–75th percentiles); whiskers extend to the minimum and maximum values within 1.5× of the interquartile range. Different uppercase letters within a zone indicate significant difference between those groups (Kruskal–Wallis test with Dunn’s multiple comparisons test, *p* < 0.05).

**Individual behavior assay.** A Kruskal–Wallis test indicated that there was no difference in total distance traveled among CTRL, BA, and THX groups (*χ*^2^ = 0.14, *df* = 2, *p* = 0.9314). However, Kruskal–Wallis analyses with the total duration spent in light or dark zone of the arena indicated that there was at least one pair of groups with a statistically significant difference in their distributions of total cumulative time spent in the particular zone of the arena (*χ*^2^ = 13.49, *df* = 2, *p* = 0.0012 for the light zone; *χ*^2^ = 10.70, *df* = 2, *p* = 0.0048 for the dark zone). Dunn’s multiple comparisons test showed that ants treated with THX spent significantly more time in the light zone (313.5 ± 8.6 s, mean ± SEM) than control ants (264.7 ± 8.6 s) while the BA-treated group (273.3 ± 10.3 s) did not differ significantly from the CTRL group in total duration spent in the light zone (Figure 6). Similarly, the ants treated with THX spent significantly less time in the dark zone (283.5 ± 8.2 s) than the CTRL group (325.9 ± 8.7 s) while the BA-treated group (303.0 ± 7.8 s) did not differ significantly from the CTRL group in total duration spent in the dark zone.

## 4. Discussion

**Figure 7:**
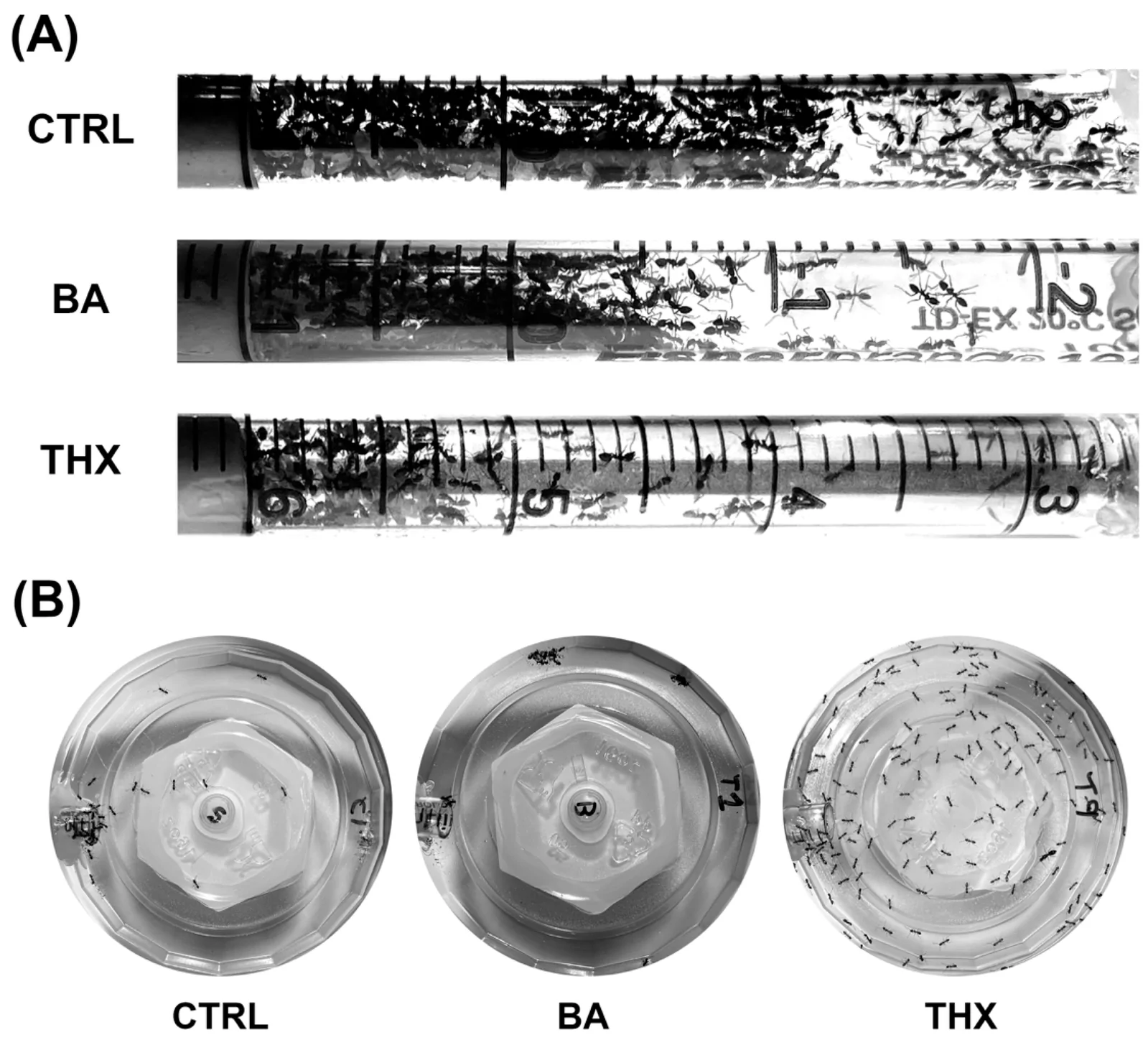
Representative photographs showing the experimental colonies on day 3 post-treatment. (**A**) Side-view images showing the nesting tube. (**B**) Top-view images showing the foraging area bottom of experimental colonies.

Our study demonstrates sublethal behavioral effects of boric acid and thiamethoxam baits on Argentine ant colonies during the initial 3 days after bait consumption. At the colony level, in-nest aggregation behavior and out-nest activity were clearly affected by these insecticides, and behavioral outcomes were markedly different between boric acid and thiamethoxam treatments. Boric acid bait treatment resulted in a significant increase in ant density within the nest (by 25.6%) and a reduced activity outside of the nest (by 56.3%) over time. In contrast, thiamethoxam bait treatment had an opposite effect, leading to a significant reduction in ant density within the nest (by 36.5%) and an increased activity outside of the nest (by 862.3%) over time. These data were corroborated by visual observation of the BA colonies on day 3, where many ants were found tightly aggregated within the nesting tube (Figure 7A, BA), while only a few ants were found in the foraging area (Figure 7B, BA). For the THX colonies, many ants were found in the foraging area (Figure 7B, THX) while the nesting tube was less crowded (Figure 7A, THX).

When the in-nest ant number data were compared across different dates (day 0–3) within a treatment, the CTRL group did not show any change over time. Also, nest volume occupied by the aggregation in the CTRL group stayed similar during this period. However, in the BA treatment, the number of ants within the in-nest aggregation increased over time (by 14.2%) (Table 2) while the nest volume (mL) occupied by in-nest aggregation stayed similar (Table 3). This indicates that the in-nest density increase in the BA group was primarily due to the increase in number of ants within an aggregation occupying the same volumetric space. In the THX treatment, the overall number of ants within the in-nest aggregation decreased (by 66.5%) (Table 2) and the nest volume occupied by in-nest aggregation was also significantly decreased during the same period (by 48.4%) (Table 3).

These contrasting results are likely due to fundamental differences between boric acid and thiamethoxam in their physiological impacts on Argentine ants. Ingested boric acid impacts insects primarily through disruption of the epithelial cells of midgut [24,26] and Malpighian tubules [26], which are associated with absorption and excretion, respectively. Increased density (also increased number) of ants in the in-nest aggregation might be due to boric acid’s toxic effect on the alimentary system and resulting impact on foraging activity [10]. Klotz and Moss [27] speculated that boric acid bait disrupts water regulation of Florida carpenter ants, causing them to produce liquid fecal matters more frequently. Thus, increased density of ant aggregation within the nest could be a result of behavioral responses of Argentine ants to conserve moisture in response to excessive water loss [34]. Besides its damage on the midgut and other parts of the alimentary tract, boric acid is known to impact other physiological processes in insects, including the cellular metabolism [35] and nervous system [24]. Therefore, it is also possible that observed in-nest aggregation and low out-nest activity of BA-treated Argentine ants could be a result of these multiple physiological impacts. Based on a behavioral observation, Zanola et al. suggested that consumption of 3% (wt/wt) boric acid in sugar water caused apparent “malaise” in Argentine ant foragers, significantly reducing overall level of motility (i.e., movement) 3 h post-ingestion [36]. Contrary to current findings in Argentine ants, an early behavioral symptom of boric acid poisoning in German cockroach, *Blattella germanica* L., was an increase in wandering outside their harborages (along with erratic movement and disorientation) [37].

On the other hand, thiamethoxam is a neurotoxin, which acts by binding to insect nicotinic acetylcholine receptors (nAChRs), causing neuronal overstimulation [14,38]. Numerous studies have demonstrated various sublethal impacts of neonicotinoid exposure in ants, such as changes in food consumption, activity and aggression level, brood care activity and colony growth, etc. [21]. In *Lasius niger* (L.) and *L. flavus* (F.), consumption of 20% honey solution containing 1.25–1.5 μg/mL imidacloprid for 3 weeks resulted in reduced overall activity, with more ants staying in their nesting tubes when the nest lids were opened [39]. However, the behavioral effect of a sublethal amount of thiamethoxam appears to be dependent upon the time after exposure. For example, consumption of honey water containing thiamethoxam (0.000028%) quickly (within 1–2 h) resulted in hyperactivity (abnormally high activity) of treated stingless bees, with increases in mean speed (~4.5-fold) and distance traveled (~5.0-fold) over the control group. In *Apis mellifera* L., consumption of thiamethoxam (1.34 ng) caused hyperactivity shortly after exposure (30 min), but this effect disappeared over a longer period (60 min) [40]. Reduced density in the in-nest aggregation (along with a reduced number of ants in the aggregation and reduced in-nest volume occupied by the aggregation) suggests that consumption of thiamethoxam bait (0.00005%) resulted in elevated activity outside of the nest and reduced nest cohesion in Argentine ant colonies.

Total travel distance data from the individual behavior assay suggested that neither BA nor THX treatment impacted the mobility of Argentine ants at the individual level (at least up to day 3 after treatment). The ants from the CTRL group and both insecticide treatment groups walked similar distances (819–848 cm on average) during the 10 min observation. This finding indicates that any impact of boric acid and thiamethoxam bait on individual ant mobility was not the driving factor for the behavioral differences observed in the earlier assays (i.e., differences found between BA and THX in density of in-nest aggregation and out-nest activity level). However, the total duration spent in the light zone was significantly longer in the THX group than the CTRL group (by 18.5%). Similarly, the total duration spent in the dark zone was significantly shorter in the THX group than the CTRL group (by 13.0%). This observation suggests that one of the behavioral effects of thiamethoxam was through the modification of the ants’ response to light. Thiamethoxam exposure may disrupt normal light-avoidance behavior [41] or increase movement toward illuminated areas. This effect is consistent with previous evidence that neonicotinoids alter insect phototaxis and locomotor activity [40], which may contribute to the broad-ranging wandering behavior observed after THX exposure. In contrast, boric acid treatment did not impact phototactic response of Argentine ants under the experimental conditions used in the current study. Thus, forming tight aggregations in the nest and low out-nest activity found in the BA-treated group could not be attributed to boric acid’s impact on phototaxis.

Our findings may provide some useful insights for practical pest ant management in urban settings. First, boric acid baiting can cause a sudden impact on foraging activity of Argentine ant colonies. The current study indicates that the exposure to diluted boric acid liquid bait (i.e., diluted through trophallaxis) could cause the affected ants to cluster together in the nest instead of leaving the nest for foraging. Overall out-nest activity of the colony would be significantly suppressed. These behavioral alterations (in addition to mortality) might be in part responsible for the quick initial suppression of Argentine ant foraging activity observed in the field after boric acid bait application [13,30,42]. However, this behavioral effect, combined with the slow action of boric acid, may allow ants to avoid further exposure to the toxicant. If some ants can recover from this initial behavioral impact and subsequently return to normal out-nest foraging activity, this could potentially contribute to a “brief recovery” after the initial baiting (within 1–4 weeks) which has been observed in some field trials with boric acid baiting [13,43,44]. Of course, other factors such as new colonies immigrating into the previously treated areas and the presence of newly emerged workers could also contribute to the resurgence [45].

On the other hand, exposure to small amounts of thiamethoxam caused Argentine ant foragers to leave the nest and increase their activity outside of the nest. Even though many of these ants with behavioral symptoms will be eventually killed within a week or so, our behavioral observation suggests that these affected foragers would be able to consume sugar water as well as water when provided [23]. Therefore, heightened activity outside of the nest could lead to greater bait consumption and potentially further toxicant spread within the colony. Simultaneously, the increased presence of “roaming” ants outside the nests could be perceived as “an increased level of ant infestation”. In practical pest management settings, the increased activity of affected ants in the well-lit locations could lead to unintended spread and, possibly, new infestations at different sites (e.g., outside building surface, windows, higher floor of the building, etc.). To combat this, cracks and openings in doors and windows could be checked and treated with a targeted application of residual insecticides (sprays and dusts) to prevent accidental introduction of the affected ants. For pest management professionals, this information will also be useful to inform their customers.

To build upon the current findings, future research could extend the behavioral observations beyond 3 days post-treatment. This would help to examine whether the observed behavioral shifts after boric acid and thiamethoxam consumption intensify, persist, or fade over time. It would also be useful to know whether some of the treated ants can recover to the control level of activity. Testing with a mixture of boric acid and thiamethoxam would be another possible direction of future research, potentially revealing the combined effect of these two different toxicants on Argentine ant colony behavior. Other common bait toxicants for ants (e.g., imidacloprid, indoxacarb, fipronil, and spinosad) could be explored for their sublethal behavioral impacts on Argentine ants. In any case, further knowledge on the behavioral impact of some of these common bait toxicants will provide useful insights for practical pest ant management and future bait product development.

## Figures and Tables

**Table 1:** Mean (±SEM) density (ants/mL) of Argentine ant workers within the nest across the experimental period. Data for day 0 were collected immediately before the bait application. Different uppercase letters within a row indicate significant differences over time (Friedman test with Dunn’s multiple comparisons test, *p* < 0.05).

Treatment	Day 0	Day 1	Day 2	Day 3
CTRL (*n* = 10)	117.4 ± 6.5 AB	108.7 ± 6.9 B	119.6 ± 9.2 AB	123.5 ± 7.3 A
BA (*n* = 10)	99.4 ± 4.4 A	100.8 ± 4.1 A	117.6 ± 4.1 B	124.8 ± 4.9 B
THX (*n* = 10)	110.9 ± 4.5 A	90.4 ± 5.5 AB	80.2 ± 4.0 BC	70.4 ± 4.9 C

**Table 2:** Mean (±SEM) counts of Argentine ant workers within aggregation in the nesting tube across the experimental period. Data for day 0 were collected immediately before the bait application. Different uppercase letters within a row indicate significant differences over time (Friedman test with Dunn’s multiple comparisons test, *p* < 0.05).

Treatment	Day 0	Day 1	Day 2	Day 3
CTRL (*n* = 10)	281.7 ± 30.5 A	285.3 ± 29.2 A	278.3 ± 27.7 A	276.5 ± 29.6 A
BA (*n* = 10)	189.6 ± 8.6 A	207.0 ± 10.7 AB	214.1 ± 11.8 B	216.6 ± 10.4 B
THX (*n* = 10)	269.0 ± 15.2 A	144.3 ± 12.7 AB	107.4 ± 7.0 BC	90.0 ± 8.5 C

**Table 3:** Mean (±SEM) volume (mL) occupied by Argentine ant workers in nesting tubes across four days following exposure to treatments. Data for day 0 were collected immediately before the bait application. Different uppercase letters within a row indicate significant differences over time (Friedman test with Dunn’s multiple comparisons test, *p* < 0.05).

Treatment	Day 0	Day 1	Day 2	Day 3
CTRL (*n* = 10)	2.44 ± 0.27 A	2.61 ± 0.20 A	2.33 ± 0.16 A	2.26 ± 0.23 A
BA (*n* = 10)	1.93 ± 0.08 AB	2.07 ± 0.10 A	1.82 ± 0.09 B	1.74 ± 0.08 B
THX (*n* = 10)	2.46 ± 0.18 A	1.61 ± 0.13 AB	1.35 ± 0.08 BC	1.27 ± 0.07 C

**Table 4:** Mean (±SEM) number of Argentine ant workers in the foraging area following exposure to treatments on day 0. Data for day 0 were collected immediately before the bait application. Different uppercase letters within a row indicate significant differences over time (Friedman test with Dunn’s multiple comparisons test, *p* < 0.05).

Treatment	Day 0	Day 1	Day 2	Day 3
CTRL (*n* = 10)	14.0 ± 2.5 A	16.4 ± 1.9 A	16.2 ± 1.7 A	17.1 ± 2.8 A
BA (*n* = 10)	17.4 ± 2.6 A	10.4 ± 1.8 B	8.8 ± 2.4 B	7.6 ± 1.1 B
THX (*n* = 10)	19.9 ± 3.0 A	121.7 ± 13.5 AB	169.1 ± 16.8 BC	191.5 ± 18.8 C

## Data Availability

The raw data supporting the conclusions of this article will be made available by the authors on request.

## Abbreviations

The following abbreviations are used in this manuscript:

CTRL	Control
BA	Boric acid
THX	Thiamethoxam
